# Clinical Outcomes of Intradiscal Condoliase Injection for Lumbar Disc Herniation: A Retrospective Analysis of the First 100 Cases

**DOI:** 10.7759/cureus.103973

**Published:** 2026-02-20

**Authors:** Atsushi Kojima, Naoki Tsujishima, Shigeru Kamitani, Hirohito Suzuki, Tomonori Sodeyama, Kenji Hatakeyama, Masao Koda

**Affiliations:** 1 Spine and Spinai Cord Center, Funabashi Orthopaedic Hospital, Funabashi, JPN

**Keywords:** chemonucleolysis, condoliase, disc degeneration, lumbar disc herniation, minimally invasive treatment

## Abstract

Background

Intradiscal injection of condoliase is a minimally invasive treatment positioned between conservative management and surgery for lumbar disc herniation (LDH).

Objective

To evaluate the short- to mid-term clinical course and radiological changes following condoliase treatment in routine clinical practice.

Methods

This retrospective observational study analyzed 100 consecutive patients with LDH who underwent intradiscal condoliase injection between December 2018 and July 2023. Clinical outcomes were evaluated using the visual analog scale (VAS). Radiological findings were assessed using established magnetic resonance imaging classifications. Clinical outcomes and subsequent treatments were analyzed.

Results

The median symptom duration was 20 weeks. Mean leg pain VAS significantly improved from 7.4 ± 2.1 at baseline to 3.7 ± 2.4 at one month and 2.4 ± 2.2 at three months (p < 0.05). Symptomatic improvement was observed in 87 patients (87.0%), with a median time to relief of four weeks. Five patients (5.0%) required subsequent surgery. No major complications were observed, including neurological deterioration, infection (discitis), anaphylaxis, hospitalization related to the procedure, or death.

Conclusions

Intradiscal condoliase injection was associated with a significant early reduction in leg pain in the majority of patients with LDH and may serve as an effective intermediate treatment between conservative management and surgery.

## Introduction

Symptomatic lumbar disc herniation (LDH) commonly presents with radicular leg pain and may be accompanied by low back pain [1]. In standard clinical management of symptomatic LDH, conservative management is typically selected as the initial treatment approach; however, some patients continue to experience persistent symptoms despite adequate nonoperative care.

Intradiscal administration of condoliase is used as a minimally invasive therapeutic option in selected patients with symptomatic LDH [2,3]. This enzymatic treatment reduces the proteoglycan content of the nucleus pulposus and has demonstrated clinical efficacy in randomized controlled trials and dose-finding studies [2,3]. Subsequent observational studies have also reported favorable outcomes in real-world clinical settings [4-6].

Radiological characteristics have been reported to correlate with clinical presentation and treatment outcomes in patients with LDH [7-9]. Magnetic resonance imaging-based classifications, including vertebral endplate changes and disc degeneration grading, provide a standardized framework for evaluating baseline pathology. Baseline MRI findings provide a standardized framework for evaluating structural pathology. Imaging changes after condoliase injection and potential predictors of clinical response have been discussed in previous reports [10,11].

The purpose of this study was to evaluate early clinical outcomes in the first 100 consecutive patients treated with intradiscal condoliase injection at our institution and to clarify the clinical role of this therapy within the current treatment strategy for LDH.

## Materials and methods

Study design and ethics

This retrospective observational study evaluated the clinical outcomes of intradiscal condoliase injection for LDH. The study was conducted in accordance with the Declaration of Helsinki. Ethical approval was obtained from the institutional review board (IRB No. 2019037). Although the protocol title differs from the current manuscript title, both refer to the same study population and investigation.

Study population

A total of 100 consecutive patients with symptomatic LDH who underwent intradiscal condoliase injection at our institution between December 2018 and July 2023 were included. Intradiscal condoliase injection refers to percutaneous injection of 1.25 units of condoliase dissolved in 1 mL solution into the nucleus pulposus under fluoroscopic guidance.

Inclusion criteria were as follows: persistent low back and/or leg pain refractory to conservative treatment for at least six weeks, imaging-confirmed LDH corresponding to clinical symptoms, and eligibility for intradiscal condoliase injection based on institutional indications.

Exclusion criteria were prior lumbar fusion surgery at the affected level, spinal infection, a tumor, a fracture, severe spinal instability requiring immediate surgical intervention, and incomplete clinical or imaging follow-up data.

Intradiscal condoliase injection procedure

Intradiscal injection was performed using a standardized technique. All procedures were conducted under fluoroscopic guidance via a posterolateral approach. Intradiscal placement was achieved based on fluoroscopic anatomical landmarks. Formal discography or contrast injection was not performed, reflecting routine clinical practice.

A single dose of condoliase (1.25 U) was injected into the nucleus pulposus. The enzyme solution was prepared according to the manufacturer's instructions and obtained from a commercially available product approved for clinical use in Japan. No additional agents or compounds were injected concomitantly.

Peri-procedural and post-procedure management

Peri-procedural medication management was standardized, and no changes were made to baseline analgesic regimens at the time of injection.

After completion of the injection, patients remained on bed rest for one hour with monitoring of vital signs. All patients were admitted for overnight observation and discharged the following morning if no adverse events were observed. A ready-made lumbar brace was prescribed for one week, and patients were instructed to avoid activities that could place excessive mechanical stress on the lumbar spine.

Data collection

Clinical and radiological data were retrospectively collected from electronic medical records. Baseline characteristics included age, sex, affected disc level, and magnetic resonance imaging findings.

Outcome measures

Pain severity was assessed using the visual analog scale (VAS), a validated tool for pain measurement [7]. Symptomatic improvement was defined as a clinically meaningful reduction in pain compared with baseline.

Magnetic resonance imaging findings were evaluated using the Modic classification for vertebral endplate changes [5] and the Pfirrmann grading system for disc degeneration [6].

Statistical analysis

Continuous variables are presented as means with standard deviations, and categorical variables are presented as numbers and percentages (N (%)). Changes in leg pain VAS scores over time (baseline, one month, and three months) were analyzed using a repeated-measures analysis of variance (ANOVA). When a significant overall effect was detected, post-hoc pairwise comparisons with Bonferroni correction were performed.

P-values were explicitly calculated and reported in the Results section, and a p-value < 0.05 was considered statistically significant. All statistical analyses were performed using standard statistical software.

## Results

The baseline patient characteristics are summarized in Table 1. The affected disc levels were L1/2 in three patients (3.0%), L2/3 in five patients (5.0%), L3/4 in 11 patients (11.0%), L4/5 in 70 patients (70.0%), and L5/S1 in 13 patients (13.0%).

**Table 1:** Baseline patient characteristics (N = 100)

Variable	Value
Number of patients	100
Mean age (years)	43.7
Sex (M/F)	65/35
Median symptom duration (weeks)	20
Affected disc level	
L1/2	3 (3.0%)
L2/3	5 (5.0%)
L3/4	11 (11.0%)
L4/5	70 (70.0%)
L5/S1	13 (13.0%)
Modic change present	14 (14.0%)
Type 1	4 (4.0%)
Type 2	9 (9.0%)
Type 3	1 (1.0%)
Pfirrmann grade ≥ IV	
Grade 1	0 (0.0%)
Grade 2	27 (27.0%)
Grade 3	66 (66.0%)
Grade 4	7 (7.0%)
Grade 5	0 (0.0%)

The clinical outcomes after intradiscal condoliase injection are summarized in Table 2. Symptomatic improvement was achieved in 87 patients (87.0%). Ten patients (10.0%) showed no improvement or worsening of symptoms, and five patients (5.0%) required subsequent surgical treatment.

**Table 2:** Clinical outcomes after condoliase injection (N = 100)

Outcome	Value
Symptomatic improvement	87 (87.0%)
No improvement or worsening	13 (13.0%)
Subsequent surgical treatment	5 (5.0%)
Median time to symptom relief	4 Weeks
Pfirrmann grade progression ≥1	51 Cases (51.0%)
Modic change after treatment	6 Cases (7.0%)

**Figure 1:**
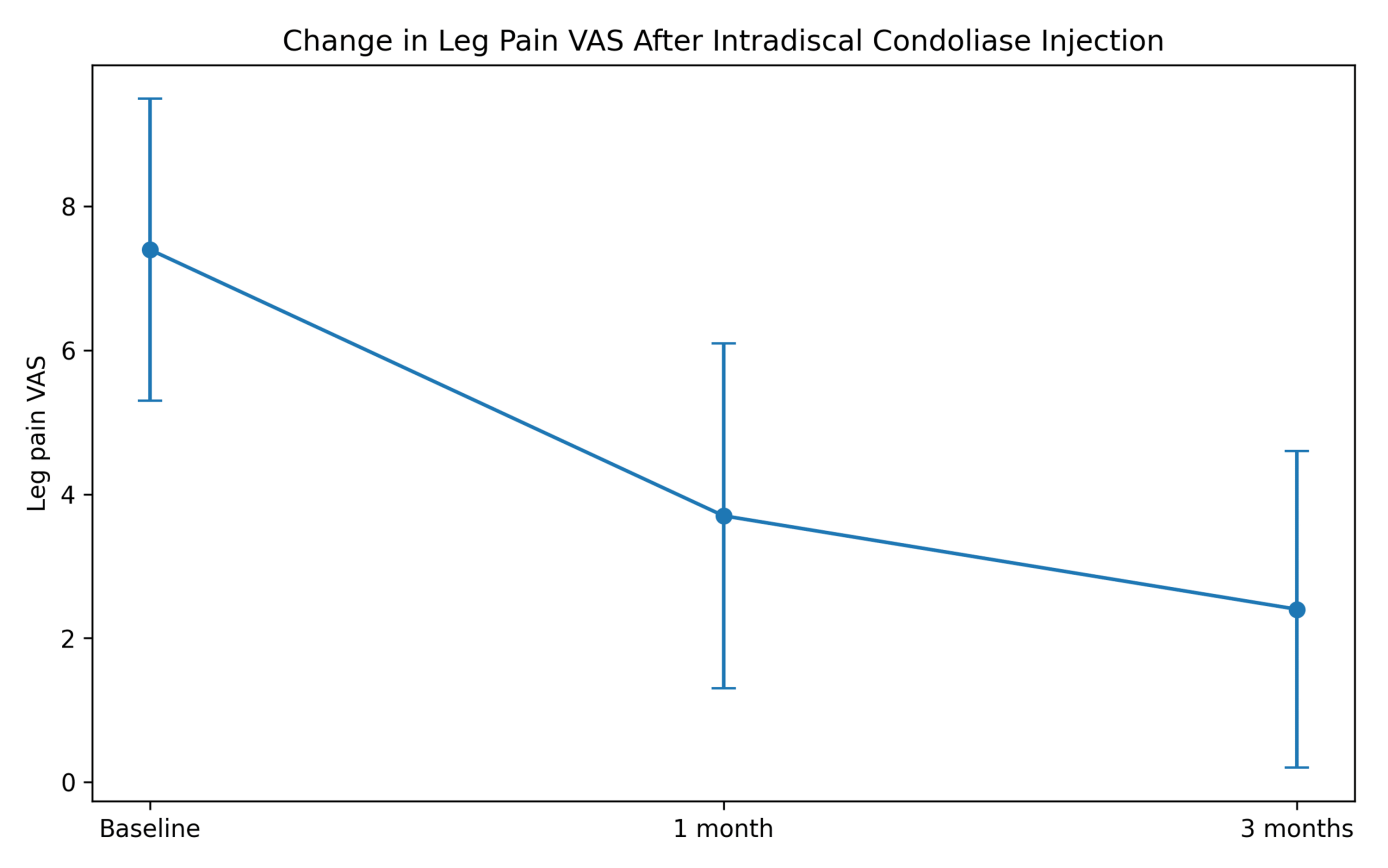
Change in leg pain VAS scores over time after intradiscal condoliase injection The mean leg pain visual analog scale (VAS) significantly improved from 7.4 ± 2.1 at baseline to 3.7 ± 2.4 at one month and 2.4 ± 2.2 at three months. Error bars represent the standard deviations.

The longitudinal changes in leg pain VAS scores demonstrated significant improvement compared with baseline (Figure 1).

The mean leg pain VAS scores showed a significant improvement over time. VAS scores decreased from 7.4 ± 2.1 at baseline to 3.7 ± 2.4 at one month and 2.4 ± 2.2 at three months. The overall change was statistically significant (repeated-measures ANOVA, p < 0.001).

Although some outcome measures demonstrated overlapping error bars, the overall trend indicated clinical improvement in approximately half of the patients. A small proportion of patients required subsequent surgical intervention during the follow-up period.

## Discussion

In this retrospective analysis of 100 consecutive patients, intradiscal condoliase injection resulted in clinically meaningful pain reduction in a substantial proportion of patients with lumbar disc herniation. This level of improvement represents a realistic and clinically relevant expectation for patients undergoing minimally invasive treatment. These findings are consistent with previous randomized controlled trials and dose-finding studies [2,3], as well as subsequent real-world clinical investigations [4,8,10,11].

Only a small proportion of patients required subsequent surgical intervention. Such cases should not be regarded as treatment failures; rather, intradiscal condoliase injection may serve as an effective intermediate treatment between prolonged conservative management and surgery. This observation is clinically relevant when considered in the context of established evidence comparing surgical and nonoperative treatment strategies for lumbar disc herniation [9,12].

Radiological characteristics may influence treatment outcomes. Previous studies have reported imaging changes following condoliase injection and have suggested potential predictive factors for treatment response [13-16]. In the present study, established MRI classification systems, including Pfirrmann grading, were used to characterize baseline pathology, providing a standardized framework for future analyses rather than for outcome prediction.

Recurrent lumbar disc herniation represents a challenging clinical scenario. Prior reports indicate that intradiscal condoliase injection may still be effective in selected recurrent cases, although careful patient selection remains essential [10].

Several limitations should be acknowledged. This study is limited by its retrospective design, single-center setting, and relatively short follow-up duration. Although overlapping error bars were observed in some outcome measures, the statistically significant improvement in mean VAS scores over time supports a consistent trend toward clinically meaningful improvement in this patient cohort. In addition, the absence of a control group precludes direct comparison with alternative treatment strategies. Despite these limitations, this study provides meaningful real-world evidence regarding early clinical outcomes after intradiscal condoliase injection in a large consecutive patient cohort.

## Conclusions

Intradiscal condoliase injection demonstrated favorable early clinical outcomes in this series of 100 consecutive patients with lumbar disc herniation. Most patients achieved symptom relief, and only a small proportion required subsequent surgical intervention. These results support the role of intradiscal condoliase injection as a minimally invasive intermediate treatment option between prolonged conservative management and surgical intervention in appropriately selected patients.
